# Ferroptosis as a potential therapeutic target for post-traumatic stress disorder

**DOI:** 10.3389/fnmol.2025.1648047

**Published:** 2025-10-23

**Authors:** Qian Zhang, Jin-Dong Mao, Hui Chen, Min Wang, Yu-Mei Wu, Chuan Wang

**Affiliations:** ^1^Department of Pharmacy, Shaanxi University of Chinese Medicine, Xianyang, Shaanxi, China; ^2^Department of Pharmacology, School of Pharmacy, Air Force Medical University, Xi’an, Shaanxi, China

**Keywords:** post-traumatic stress disorder, ferroptosis, lipid peroxidation, iron dysregulation, inflammatory responses, therapeutic target

## Abstract

The underlying mechanisms of post-traumatic stress disorder (PTSD) are still not fully understood, creating significant obstacles for developing effective therapeutic strategies. Recently, ferroptosis, an iron-dependent form of regulated cell death, has been shown to play a role in several psychiatric disorders, such as major depressive disorder (MDD), stress-induced anxiety, Alzheimer’s disease (AD), and Parkinson’s disease (PD). While direct evidence for the role of ferroptosis in PTSD is still limited, an increasing number of studies suggest that the pathological features of PTSD may trigger the ferroptosis cascade. Additionally, the typical hallmarks of ferroptosis, such as iron dysregulation, lipid peroxidation, and failure of antioxidant defense systems, may intersect with the pathogenesis of PTSD. Importantly, some treatments for PTSD, such as antioxidants and free radical scavengers, have been proven to inhibit ferroptosis, which further supports the case for ferroptosis as a potential pathogenic mechanism in PTSD. To thoroughly investigate the mechanistic links between ferroptosis and PTSD, we analyze the relevant literature on ferroptosis and PTSD in this review. Our aim is to elucidate the potential relationships between ferroptosis and PTSD, thereby providing novel insights for future research directions. Furthermore, we call for more experimental and clinical studies to explore this relationship further, with the ultimate goal of developing more effective therapeutic strategies for PTSD.

## Graphical Abstract

**Figure:**
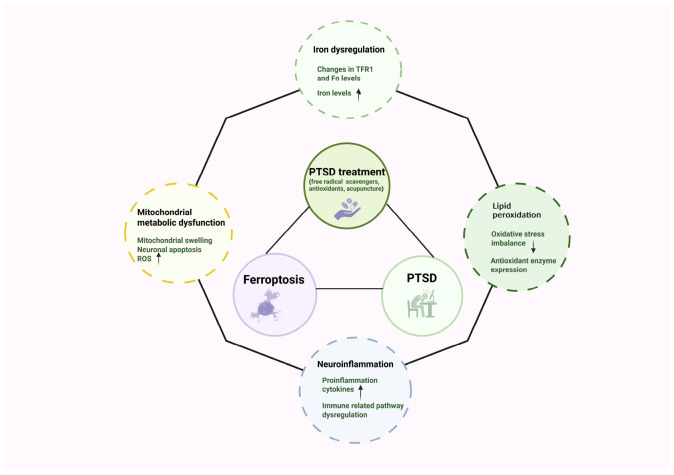
The potential connections between ferroptosis and PTSD. It illustrates the connections between the two, including iron dysregulation, lipid peroxidation, neuroinflammation, and mitochondrial metabolic dysfunction. Each element includes related biochemical changes and effects, such as oxidative stress and neuron apoptosis. Additionally, it explores some potential therapeutic strategies targeting ferroptosis in PTSD, encompassing free radical scavengers, antioxidants, and acupuncture.

## 1 Introduction

Post-traumatic stress disorder (PTSD) is a psychiatric condition characterized by the re-experiencing of traumatic events, heightened vigilance, anxiety, depression, and other psychological challenges, all of which significantly impact patients’ mental health (Ressler et al., 2022). Research has confirmed that the prevalence of PTSD ranges from 1 to 8% in the general population and rises to 50% in mental health institutions (Maercker et al., 2022). Notably, more than one-third of PTSD patients suffer from chronic and treatment-resistant symptoms, often due to delayed diagnoses. The pathogenesis of PTSD is extremely complex and it involves multiple biological processes, including inflammatory responses, alterations in neuroplasticity, and dysregulation of neurotransmitter systems (Zhang et al., 2014; Hori and Kim, 2019; López-López and Crespo, 2025). Notably, lipid peroxidation dysfunction, a hallmark of ferroptosis, and alterations in lipid-related cascades have been identified as crucial factors in the pathogenesis of PTSD (Atli et al., 2016; Pope and Dixon, 2023). Additionally, the changes in the function, structure, and chemical processes of fear learning and memory circuits are closely associated with the development of PTSD and also pose considerable challenges for its treatment (Harnett et al., 2020). Given the limited efficacy of existing pharmacological treatments for PTSD, there is an urgent need for further research to uncover the underlying mechanisms of PTSD and identify new therapeutic targets.

## 2 Overview of ferroptosis

Ferroptosis, a distinctive form of regulated cell death (RCD), was first defined by Dixon et al. (2012). Morphologically, ferroptosis is quite different from necrosis, apoptosis, and autophagy on cellular morphology and function. It lacks the characteristic features of classical necrosis, including cytoplasmic and organelle swelling, as well as rupture of the plasma membrane. Similarly, it does not exhibit features of traditional apoptosis, including cell shrinkage, chromatin condensation, apoptotic body formation, and cytoskeletal disintegration. Notably, ferroptosis is primarily characterized by pronounced mitochondrial shrinkage, increased membrane density, and reduction or loss of mitochondrial cristae (Xie et al., 2016; Li et al., 2020). These morphological and ultrastructural changes are important markers that distinguish ferroptosis from other forms of RCD. Biochemically, the core mechanism of ferroptosis lies in the iron-dependent accumulation of lipid peroxides, which can be summarized as a cascade reaction of “iron overload-lipid oxidation-antioxidant failure”. Specifically, excessive redox-active iron in cells can generate reactive oxygen species (ROS) through pathways such as the Fenton reaction. These ROS preferentially attack phospholipid molecules containing polyunsaturated fatty acids (PUFAs), triggering large-scale lipid peroxidation reactions, which in turn disrupt intracellular redox homeostasis and ultimately initiate the ferroptosis process (Jiang et al., 2021). Genetically, ferroptosis is controlled by genes in multiple pathways (Xie et al., 2016; Tang et al., 2021). For example, the system Xc^–^ pathway transports cystine into cells to supply precursors for GSH synthesis. In this pathway, downregulation of pathway-related genes, such as SLC7A11, reduces GSH production and indirectly promotes ferroptosis. Despite these findings, the intricate genetic and regulatory mechanisms underlying ferroptosis remain to be fully elucidated.

**FIGURE 1:**
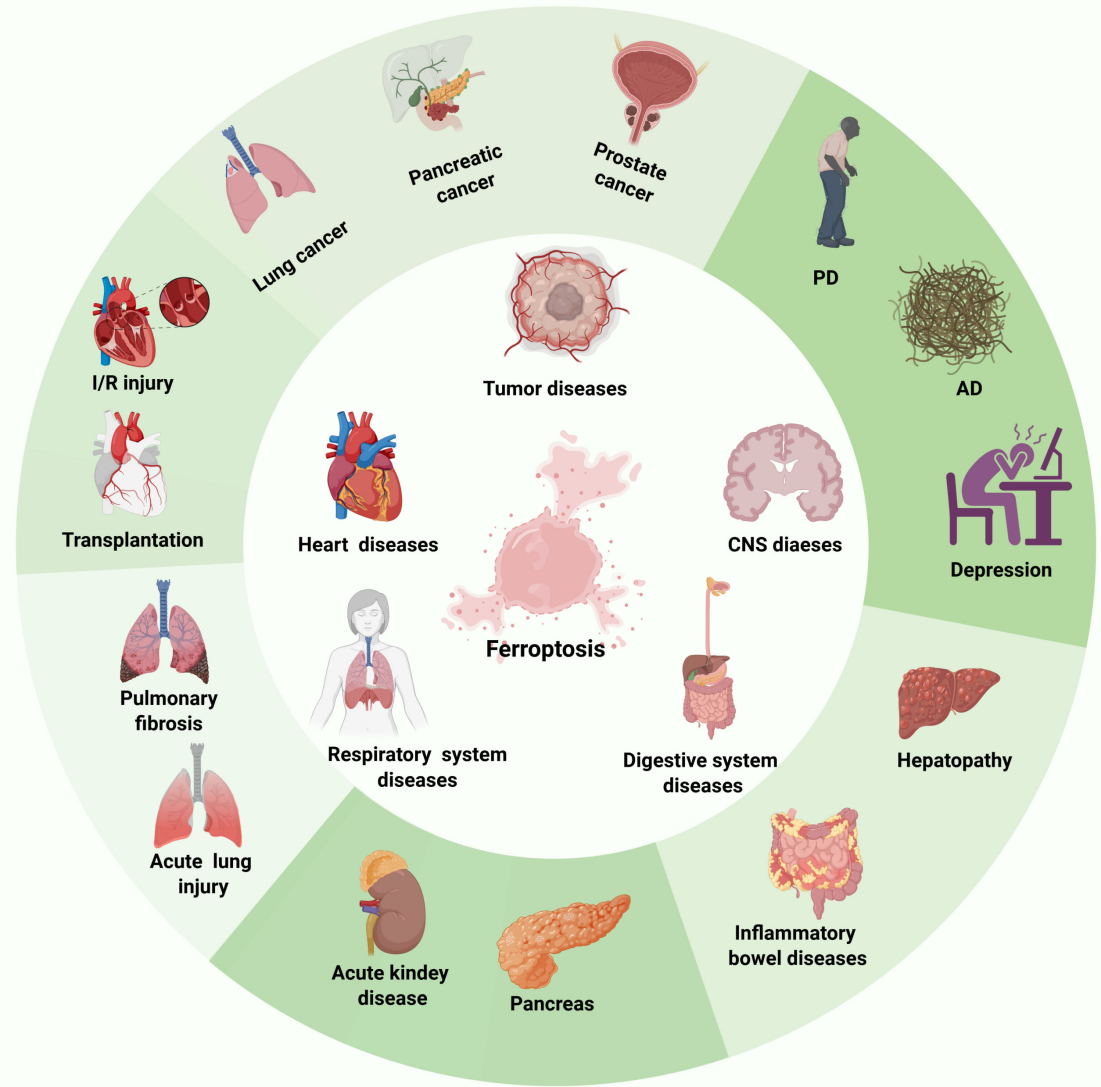
The relationship between ferroptosis and various diseases. This figure illustrates the widespread impact of ferroptosis across multiple system diseases, such as tumor diseases, CNS diseases, digestive system diseases, respiratory system diseases, heart diseases, kidney diseases, pancreatic diseases, and so on. This suggests that ferroptosis could serve as a potential therapeutic target in a wide range of diseases.

Initially identified in cancer research, ferroptosis has been shown to exert impact on tumor growth, progression, and chemotherapy resistance (Jiang et al., 2021). Accumulating evidence further indicates that ferroptosis significantly regulates the onset and progression of various other diseases (Figure 1), including many central nervous system (CNS) disorders, such as Alzheimer’s disease (AD), Parkinson’s disease (PD), and ischemic brain injury (Bao et al., 2021; Mahoney-Sánchez et al., 2021; Li C. et al., 2022; Gleason and Bush, 2021). These findings have positioned ferroptosis as a research focus and a promising therapeutic target for improving the treatment and prognosis of related diseases. While direct studies investigating the role of ferroptosis in PTSD remain limited. Notably, individuals with PTSD have been confirmed to exhibit elevated oxidative stress and disrupted iron metabolism (Zhao et al., 2016; Miller et al., 2018), which are central pathological features of ferroptosis. Moreover, postmortem human studies have further revealed altered transcription of oxidative stress-related genes in the dorsolateral prefrontal cortex (DLPFC) of PTSD patients (Watling et al., 2023). Furthermore, existing research has identified three key genes that predict the risk of developing PTSD, namely ACSL4, ACO1, and GSS. These genes are involved in regulating lipid, iron, cysteine, and glutathione metabolism and they are not only critical components of ferroptosis but also participate in its associated biological networks (Zhu et al., 2022). Collectively, this evidence suggests that ferroptosis may play a significant role in the development and progression of PTSD, potentially offering new therapeutic strategies for its treatment.

## 3 Methodology

To identify key research findings on the association between ferroptosis and PTSD in existing literature, a comprehensive literature search was conducted. First, the search employed targeted keywords and phrases to capture relevant studies, including “ferroptosis and PTSD,” “iron and PTSD,” “PTSD and oxidative stress,” and “PTSD and lipid.” Notably, these terms were specifically selected to cover core biological processes linked to ferroptosis and their potential connections to PTSD. Second, the search was expanded across three major academic databases: PubMed, Google Scholar, and Web of Science, ensuring broad coverage of both clinical and preclinical research. After screening for relevance and direct evidence of the ferroptosis-PTSD relationship, a total of 15 articles were included in this review (Table 1). The ultimate goal of this study is to synthesize the key findings from these 15 included articles, with the aim of providing foundations for advancing the novel treatment strategies for PTSD. The core objective of this study is to conduct an integrated analysis of the key research findings from these 15 included literatures, thereby providing a theoretical basis and practical foundation for the development of novel therapeutic strategies for PTSD.

**TABLE 1:** Characteristics of include studies in the review.

Publication year	Study type	Markers evaluated	Inclusion criteria	Boolean operators	Keywords	References
2003	Research study	GSH-Px, CAT, MDA, SOD	PTSD patients, ferroptosis-related markers	And	PTSD, free radicals	Tezcan et al., 2003
2015	Research study	GPX, SOD	PTSD models, ferroptosis-related markers	And	PTSD, oxidative stress	Borovac Štefanović et al., 2015
2016	Research study	TfR1, Fn	PTSD models, ferroptosis-related markers	And	PTSD, iron	Zhao et al., 2016
2016	Research study	MDA	PTSD patients, ferroptosis-related markers	And	PTSD, lipid peroxidation, oxidative stress	Atli et al., 2016
2018	Research study	GSH, GSSG, TBAR	PTSD models, ferroptosis-related markers	And	PTSD, oxidative stress	Alzoubi et al., 2018b
2018	Review	oxidative stress, inflammation	PTSD models, ferroptosis-related markers	And	PTSD, oxidative stress, inflammation	Miller et al., 2018
2019	Research study	GSH, GSSG, GPX, TBARS	PTSD models, ferroptosis-related markers	And	PTSD, oxidative stress	Alzoubi et al., 2019
2019	Research study	Nrf2, keap1, HO-1	PTSD models, ferroptosis-related markers	And	PTSD, keap1/Nrf2	Zhou et al., 2019
2020	Research study	GSH, GSSG, GPX	PTSD models, ferroptosis-related markers	And	PTSD, oxidative stress	Ahmed et al., 2020
2021	Research study	FPN1	PTSD models, ferroptosis-related markers	And	PTSD, FPN1	Wu et al., 2021
2022	Research study	PUFA	PTSD models, oxidative and lipid homeostasis are altered in PTSD models	And	PTSD, lipids	Kelley et al., 2022
2022	Research study	ACSL4, ACO1, GSS	Studies evaluating ferroptosis-related markers	And	PTSD, Ferroptosis, inflammatory pathways	Zhu et al., 2022
2023	Research study	GSH	PTSD models, ferroptosis-related markers	And	PTSD, GSH, Psychiatric Disorder	Watling et al., 2023
2023	Review	Mitochondria	PTSD models, changes related to ferroptosis	And	PTSD, inflammation, mitochondria	Kim et al., 2023
2024	Review	Lipid peroxidation	PTSD models, changes related to ferroptosis	And	PTSD, fatty acids, sphingomyelins, triglycerides	Bhargava et al., 2024

GSH-Px, glutathione peroxidase; CAT, catalase; MDA, malondialdehyde; SOD, superoxide dismutase; GPX, glutathione peroxidase; TfR1, transferrin receptor 1; Fn, ferritin; GSH, reduced glutathione; GSSG, oxidized glutathione; TBAR, thiobarbituric acid reactive substances; Nrf2, nuclear factor erythroid 2-related factor 2; FPN1, ferroportin 1; Keap1, kelch-like ECH-associated protein 1; PUFA, polyunsaturated fatty acids; ACSL4, Acyl-CoA synthetase long-chain family member 4; ACO1, Aconitase 1; GSS, glutathione synthetase.

## 4 The mechanisms of ferroptosis

**FIGURE 2:**
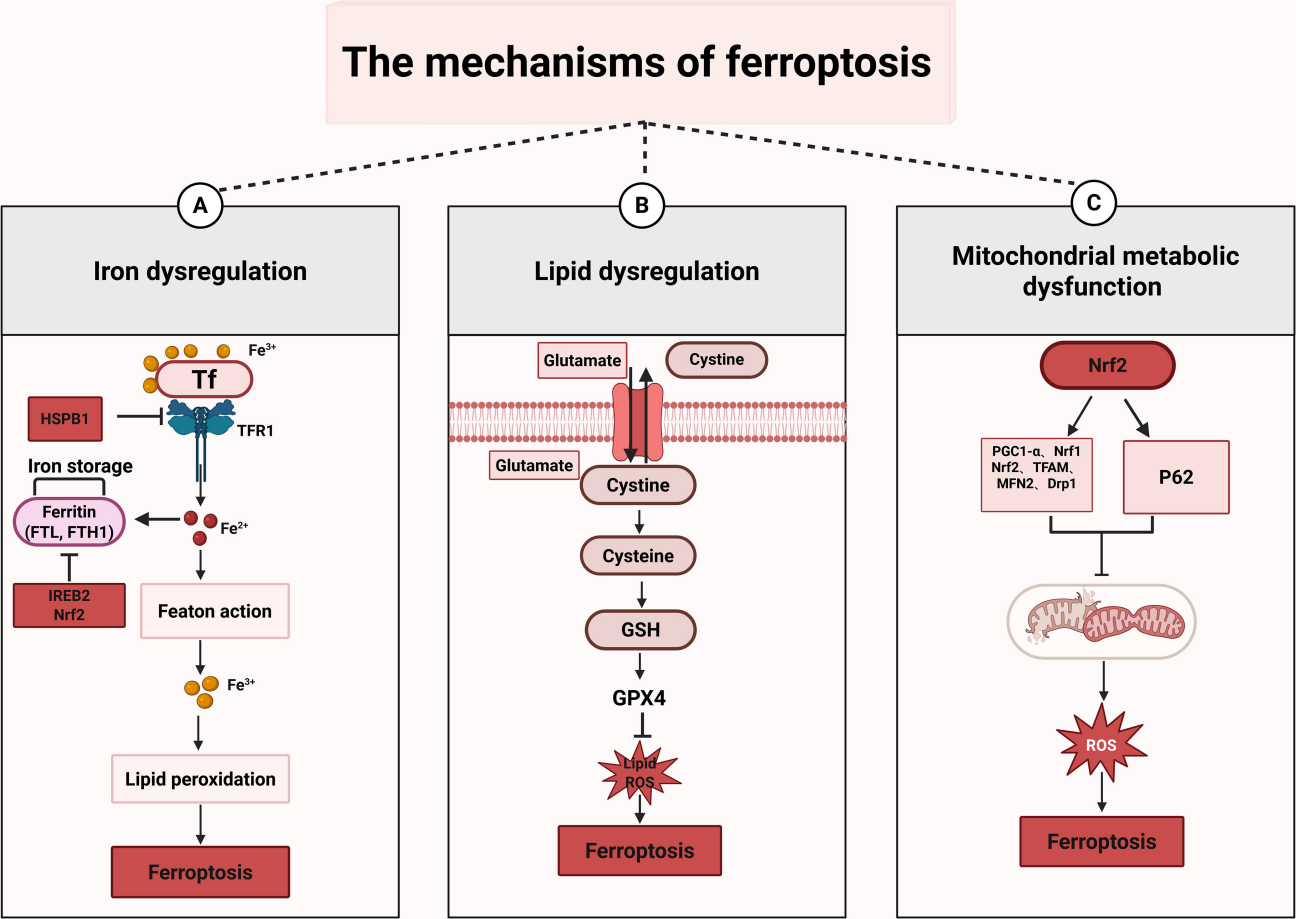
The mechanisms of ferroptosis. This figure illustrates the three primary mechanisms of ferroptosis. **(A)** Iron dysregulation: when TF binds to Fe3^+^, it enters the cell through TFR1, leading to the conversion of Fe3^+^ into Fe2^+^, which can initiate the Fenton reaction. Moreover, Fe3^+^ participates in lipid peroxidation and ultimately triggers ferroptosis. In this process, HSPB1 inhibits ferroptosis by suppressing TFR1; while IREB2 and Nrf2 can inhibit ferroptosis by inhibiting FTH1. **(B)** Lipid dysregulation: cystine is transported into cells and subsequently converted into cysteine. Cysteine then participates in the synthesis of GSH. GSH, under the action of GPX4, inhibits the generation of lipid and ROS. When the function of GPX4 is impaired, the lipid and ROS accumulate, which can trigger ferroptosis. **(C)** Mitochondrial metabolic dysfunction: Nrf2 is a transcription factor that regulates the expression of a series of genes to influence mitochondrial function, including PGC1-α, Nrf1, Nrf2 itself, TFAM, MFN2, and Drp1. When mitochondrial function is impaired, it leads to excessive production of ROS. This overabundance of ROS can damage cells and ultimately result in ferroptosis.

The regulatory mechanisms of ferroptosis are complicated, encompassing a diverse array of signaling molecules and metabolic pathways (Figure 2).

### 4.1 Iron dysregulation

Iron metabolism is a tightly regulated process that encompasses iron uptake, distribution, storage, utilization, and efflux (Gao et al., 2019). Generally, cellular iron acquisition primarily depends on the coordinated action of transferrin (TF) and transferrin receptor 1 (TFR1). Specifically, TF transports iron from storage sites to the extracellular environment, where it binds to TFR1. TFR1 is a type II transmembrane glycoprotein widely expressed in mammalian cells and is critical for iron uptake. Notably, TFR1 expression is dynamically regulated by intracellular iron levels: iron deficiency upregulates TFR1 to enhance iron uptake, while iron excess downregulates TFR1 to prevent overload (Kawabata, 2019).

Beyond the canonical pathway, multiple intracellular molecules precisely control iron metabolism to regulate ferroptosis progression. For instance, heat shock protein family B member 1 (HSPB1) lowers intracellular iron levels by downregulating TFR1, thereby inhibiting ferroptosis (Sun et al., 2015). Similarly, iron-responsive element-binding protein 2 (IREB2) maintains iron homeostasis and enhances antioxidant defense by increasing the levels of ferritin heavy chain (FTH1) and ferritin light chain (FTL), ultimately exerting an effect that resists ferroptosis (Lv et al., 2022). Furthermore, nuclear factor erythroid 2-related factor 2 (Nrf2), a core regulator of oxidative stress responses, suppresses ferroptosis through promoting FTH1 transcription, which helps maintain iron balance and improve antioxidant capacity (Qian et al., 2024). Conversely, when intracellular iron homeostasis is disrupted, excess iron generates ROS via the Fenton reaction, activates iron-dependent enzymes, induces lipid peroxidation and membrane oxidative damage, and eventually initiates ferroptosis (Dixon et al., 2012). These findings underscore that the accurate regulation of iron metabolism is vital for preserving cellular homeostasis, preventing ferroptosis, and safeguarding organismal health.

### 4.2 Lipid dysregulation

Lipid peroxidation serves as a pivotal factor in ferroptosis, occurring via both enzymatic and non-enzymatic mechanisms (Lee et al., 2021; Zuo et al., 2023). In the non-enzymatic pathway, free ferrous iron in cells triggers the Fenton reaction, which catalyzes the breakdown of hydrogen peroxide (H_2_O_2_) and produces highly reactive hydroxyl radicals (●OH). These radicals initiate lipid peroxidation by abstracting hydrogen atoms from the bis-allylic position of PUFAs, leading to the accumulation of lipid peroxides and subsequent cell membrane damage. In contrast, the enzymatic pathway relies on specific catalytic proteins (Lee et al., 2021). Lipoxygenases (ALOXs), which are key drivers of lipid peroxidation, convert PUFAs into lipid peroxides, such as arachidonic acid hydroperoxide. These peroxides increase cell membrane fluidity and permeability, disrupt cellular functions, and eventually induce ferroptosis. Evidence confirms that reducing ALOXs levels can alleviate cell death induced by ferroptosis inducers (Liu et al., 2022). Furthermore, compounds like vitamins and flavonoid-based drugs can suppress ALOX activity, acting as ferroptosis inhibitors (Shah et al., 2018). Additionally, cytochrome P450 reductase facilitates ferroptosis through the lipid peroxidation process, which begins with the peroxidation of PUFAs *via* electron transfer, a mechanism closely associated with the lipid peroxidation cascade (Feng and Stockwell, 2018). These findings underscore the crucial role of lipid peroxidation in ferroptosis.

Notably, molecules that regulate lipid peroxidation are critical in ferroptosis, as dysfunction of these regulators profoundly influences cellular fate. For instance, acyl-CoA synthetase long-chain family member 4 (ACSL4) is a critical factor that facilitates lipid peroxidation by catalyzing the esterification of PUFAs with CoA to generate PUFA-CoA. Studies have demonstrated that ACSL4 deficiency disrupts intracellular PUFA metabolism, significantly reducing lipid peroxide levels and consequently decreasing cellular susceptibility to ferroptosis (Ding et al., 2023). Importantly, ACSL4 is also linked to the risk of PTSD and regulates the metabolism of lipid, iron, cysteine, and GSH, all of which are core pathways implicated in ferroptosis (Zhu et al., 2022). Therefore, further investigation into ACSL4 and its regulatory metabolic network is essential for elucidating the mechanisms of ferroptosis and developing therapeutic strategies for related diseases. Another key mediator of ferroptosis is lysophosphatidylcholine acyltransferase 3 (LPCAT3), which plays a vital role by incorporating oxidation-sensitive PUFAs into cellular membranes. Specifically, LPCAT3 mediates the re-esterification of PUFA-CoA into membrane phospholipids, thereby providing substrate pools for lipid peroxidation. In contrast, genetic ablation or knockdown of LPCAT3 impairs this process, resulting in decreased production of membrane lipid peroxides and subsequent inhibition of ferroptosis (Liu et al., 2022). Collectively, these findings indicate that both ACSL4 and LPCAT3 are pivotal for orchestrating lipid metabolism and controlling ferroptosis.

### 4.3 Mitochondrial metabolic dysfunction

Ferroptosis, a regulated form of cell death, originates and propagates across multiple organelles, including mitochondria, endoplasmic reticulum, Golgi apparatus, and lysosomes (Gan, 2021). Among these, mitochondria play a pivotal role in cellular energy metabolism, performing key biological functions such as ATP production through oxidative phosphorylation and maintaining redox and calcium homeostasis (Yapa et al., 2021). Notably, mitochondrial energy metabolism is significantly altered during ferroptosis, with increased oxidative phosphorylation and ATP production, and a decreased glycolysis rate. Moreover, excessive oxidative stress also causes irreversible damage to mitochondrial structural. Beyond energy metabolism and structure stability, mitochondria also contribute to iron balance regulation within the nervous system. Specifically, they facilitate cell death signals induced by increased lipid peroxidation in neuronal cells (Gao and Chang, 2014). Notably, some ferroptosis inhibitors specifically target mitochondrial pathways. For instance, Cotticelli et al. (2019) has reported that mitochondrial iron overload occurs in Friedreich ataxia (FRDA), and the mitochondria-targeted antioxidant XJB-5–131 significantly reduces ferroptosis in cells. This further confirms that targeting mitochondrial pathways is a promising strategy for regulating ferroptosis.

Notably, multiple intracellular molecules regulate mitochondrial metabolism to modulate ferroptosis. Among them, the antioxidant transcription factor Nrf2 is a crucial regulator of mitochondrial function and ferroptosis process (Han et al., 2024). Specifically, Nrf2 can influence mitochondrial biogenesis by modulating the expression of molecules like PGC-1α, Nrf1 and TFAM, and mitochondrial genes. Additionally, it can regulate mitophagy through a PINK1/Parkin-independent mechanism. Nrf2 also controls mitochondrial fission and fusion processes by regulating the expression of fission-related proteasome genes and fusion-related MFN2 (Li et al., 2023). Another important molecule is AMPK, which is a critical sensor of cellular energy status and regulates an adaptive response under energy stress. Recent study has shown that energy stress activates AMPK, which inhibits acetyl-CoA carboxylase (ACC), thereby restraining fatty acid synthesis (FAS) and inhibiting ferroptosis (Lee et al., 2020). Moreover, FoxO3a activity, which is activated by the AMPK pathway, plays a unique role in the energy stress response and ROS regulation. It has been indicated that AMPK phosphorylates FoxO3a at serine 413 under energy stress. This phosphorylation enhances FoxO3a-dependent transcription and confers resistance to erastin-induced ferroptosis (Zhong et al., 2023). Therefore, a deeper understanding of the interactions and mechanisms of these molecules in ferroptosis is important for developing therapeutic strategies for related diseases.

## 5 Hallmarks of ferroptosis interact with PTSD pathogenesis

### 5.1 Iron metabolic disturbance

Significant disturbances in iron metabolism have been confirmed in PTSD. Existing evidence has confirmed that single prolonged stress (SPS), a common PTSD model (Souza et al., 2017), significantly elevates plasma cortisol levels and increases iron content in critical brain areas, such as the hippocampus, prefrontal cortex, and striatum. Meanwhile, stress also induces region-specific changes in both protein and mRNA levels of transferrin receptor 1 (TfR1) and ferritin (Fn). Notably, fn serves as an important protein responsible for the iron storage and iron detoxification (Joshi and Clauberg, 1988). These findings suggest that iron metabolism abnormalities may be a key factor in the pathogenesis and development of PTSD. Further research has revealed that brain areas with iron accumulation exhibit mitochondrial swelling and neuronal apoptosis (Zhao et al., 2016). Given that mitochondrial dysfunction and lipid peroxidation are hallmarks of ferroptosis, these findings suggest that iron accumulation in specific brain regions may not only disrupt normal cellular functions but also potentially trigger ferroptosis.

Notably, the pathophysiology of PTSD is closely linked to the dysregulation of multiple neurotransmitter systems, including glutamate, norepinephrine and dopamine (Zhang et al., 2014). Specifically, PTSD patients often exhibit persistent activation of the hypothalamic-pituitary-adrenal (HPA) axis, which not only enhances excitatory glutamatergic neurotransmission but also leads to hyperactivity of the noradrenergic system (Nutt, 2000). These abnormalities in neurotransmitter systems are directly linked to the heightened vigilance and exaggerated fear responses observed in PTSD patients. Meanwhile, PTSD patients frequently show decreased 5-hydroxytryptamine (5-HT) function, which impairs the inhibitory control of emotions and impulsive behaviors. Additionally, alterations in dopaminergic signaling may occur in specific brain regions, such as the reward circuit, in PTSD patients (Zhang et al., 2014). Collectively, these neurochemical imbalances constitute a key mechanism underlying the core symptoms of PTSD, including hypervigilance, anxiety and traumatic re-experiencing. Notably, iron is essential for the normal function of multiple neurotransmitters, including dopamine, norepinephrine, and serotonin. Among these interactions, a significant toxic interplay exists between iron and dopamine. On one hand, dopamine can promote intracellular iron accumulation, which further triggers oxidative stress responses (Dichtl et al., 2018). On the other hand, excessive iron can exacerbate the toxic effect of specific neurotoxic metabolites generated during dopamine metabolism, thereby forming a toxic cycle (Hare and Double, 2016). Furthermore, iron and glutamate exhibit a close bidirectional regulatory relationship. Iron participates in glutamate synthesis through activating cytosolic aconitase, while glutamate enhances cellular iron uptake capacity by upregulating the expression of divalent metal transporter 1 (DMT1). This regulatory loop ultimately leads to an increase in total brain iron levels, which further disrupts the homeostasis of nerve cells (McGahan et al., 2005). Given that research has already confirmed the presence of iron metabolic disturbances in PTSD, it is plausible that disturbances in iron metabolism may interact with the dysregulation of the neurotransmitter system, thereby contributing to the development of core symptoms of PTSD. This further underscores the potential importance of iron homeostasis management in PTSD treatment. Moreover, research has demonstrated that the conditional knockout of FPN1 in mouse neurons disrupts iron homeostasis, resulting in iron deficiency in the cortex and hippocampus. This iron deficiency subsequently affects contextual fear responses in mice (Wu et al., 2021). Collectively, these findings underscore the importance of managing iron homeostasis and understanding the mechanisms of ferroptosis, which may offer innovative therapeutic strategies for addressing PTSD.

### 5.2 Lipid oxidative damage

Lipid peroxidation, a key driver of ferroptosis, has been widely recognized for its significant role in PTSD pathophysiology. Research has indicated that PTSD patients exhibit downregulated expression of GPX and pronounced oxidative stress imbalance. Specifically, decreased levels of superoxide dismutase (SOD) and GPX have been observed in the bloodstream of PTSD patients (Borovac Štefanović et al., 2015; Hassan et al., 2016). Further studies have shown that malondialdehyde (MDA), a lipid peroxidation byproduct, is significantly elevated in both military personnel and earthquakes survivors with PTSD. The degree of SOD and GPX dysfunction positively correlates with symptom severity (Tezcan et al., 2003). Beyond oxidative stress, PTSD patients also exhibit lipid metabolism abnormalities, including alterations in sphingolipids and phospholipids in both plasma and cerebrospinal fluid (Zieker et al., 2007; Atli et al., 2016; Bhargava et al., 2024). The elevation of these biomarkers is an important signal indicating the occurrence of ferroptosis. Consequently, these findings suggest that significant pathological mechanisms underlying PTSD may involve dysregulated lipid peroxidation and oxidative stress pathways, which are central to the process of ferroptosis.

Supporting evidence from animal models further indicates that traumatic stress can lead to disrupted lipid metabolism. In PTSD models, disturbances in hippocampal lipid and oxidative homeostasis have been confirmed, including elevated cholesterol levels, altered lipid profiles, increased free radical generation, and abnormal levels of oxidized polyunsaturated fatty acids (PUFAs) (Kelley et al., 2022). These changes may contribute to PTSD development and potentially trigger the ferroptosis cascade. Furthermore, elevated levels of ROS, peroxynitrite, and superoxide have been detected. Chronic ROS accumulation has been shown to impair hippocampal neurogenesis in mice deficient in SOD (Wilson et al., 2013). Moreover, excessive ROS production can lead to mitochondrial swelling and calcium leakage, thereby initiating a cycle of neuroinflammation that exacerbates PTSD symptoms (Lushchak et al., 2022; Kmita et al., 2023). Additionally, significantly increased levels of thiobarbituric acid reactive substances (TBARs), which are lipid peroxidation byproducts, have been reported in PTSD models (Alzoubi et al., 2018a). Given that ferroptosis is characterized by iron-dependent lipid peroxidation, the observed lipid metabolic disturbances and oxidative stress in PTSD patients and PTSD animal models strongly suggest that ferroptosis may be a significant pathological mechanism in PTSD.

### 5.3 Vicious circle of inflammation

Inflammation is a protective response of the body to injury or infection, which typically involves activating immune cells and releasing various cytokines. Studies have consistently demonstrated that PTSD patients exhibit higher levels of pro-inflammatory cytokines like TNF-α and IL-1β (Hori and Kim, 2019; Pivac et al., 2023). Notably, these cytokines may not only directly contribute to neuronal damage but also exacerbate ferroptosis by dysregulating iron metabolism and promoting lipid peroxidation. Moreover, lipid peroxidation products generated during ferroptosis, sch as MDA, may further stimulate inflammatory responses, creating a vicious cycle. This cycle underscores the potential for ferroptosis to perpetuate and amplify neuroinflammation in PTSD. Furthermore, increased activity of the NF-κB pathway has been observed in PTSD patients (Gupta and Guleria, 2022). Importantly, NF-κB activation triggers a cascade of signaling events, which leads to the production of pore-forming proteases, the activation of alarmins, and the stimulation of matrix metalloproteinases (MMPs). These enzymes play a crucial role in degrading both cellular and extracellular components of the blood-brain barrier (BBB), such as tight junction and adherens junction proteins. Eventually, this results in increased BBB permeability in vulnerable brain regions (Welcome and Mastorakis, 2020). Notably, iron accumulation has been confirmed in these some brain regions in PTSD models (Zhao et al., 2016). Iron overload may not only directly promote lipid peroxidation but also indirectly amplify neuroinflammatory responses via NF-κB activation, forming another deleterious feedback loop that collectively exacerbates neuronal injury and BBB disruption. Importantly, whole transcriptome analyses of peripheral blood from PTSD patients reveal broad dysregulation in immune-related pathways (Zhu et al., 2022). This suggests that ferroptosis may serve both as a consequence and a driver of immune dysfunction in PTSD.

### 5.4 Mitochondrial dysfunction

As a central regulator of cellular metabolism and death, mitochondrial play a pivotal role in ferroptosis (Gan, 2021). Specifically, mitochondrial dysfunction exacerbates oxidative stress and increases intracellular ROS levels, thereby promoting ferroptotic cell death. Additionally, mitochondrial dysfunction is frequently accompanied by significant morphological alterations, including increased mitochondrial fission and cristae enlargement, which are closely associated with increased cellular susceptibility to ferroptosis (Song et al., 2024). Notably, MitoQ, an effective mitochondrial ROS scavenger, can improve mitochondrial function by inhibiting ferroptosis (Jelinek et al., 2018), offering a promising therapeutic strategy for ferroptosis-related diseases. Further research has underscored the significance of mitochondrial dysfunction and oxidative stress in the progression of PTSD. For instance, elevated ROS levels can disrupt calcium homeostasis, leading to mitochondrial swelling and Ca^2+^ leakage. This disruption may create a vicious cycle of mutual reinforcement between ROS and Ca^2+^, potentially aggravating PTSD symptoms (Lushchak et al., 2022; Kmita et al., 2023). Additionally, disturbances in iron metabolism have been confirmed in PTSD models, which are closely intertwined with oxidative stress. However, abnormal iron accumulation may further exacerbate oxidative stress and activate ferroptosis-related pathways, thereby increasing the vulnerability of neural cells to ferroptosis. Moreover, studies have revealed that PTSD models exhibit marked mitochondrial impairment. Specifically, increased expression of fission-related proteins FIS1 and DRP1, alongside reduced levels of fusion regulators OPA1 and MFN2, have been detected in the amygdala. Transmission electron microscopy further confirms the presence of swollen and damaged mitochondria in this brain region among stressed rats (Papageorgiou and Filiou, 2024; Prajapati et al., 2024). Collectively, these findings suggest that mitochondrial alterations constitute a key pathological mechanism in PTSD, contributing to ferroptosis and disease progression.

### 5.5 Role of P53 in ferroptosis and PTSD pathophysiology

The transcription factor p53 is a tumor suppressor that regulates multiple oncogenes expression and their downstream signaling pathways, thereby mediating diverse biological effects (Liu et al., 2019; Levine, 2020). In tumor tissues, mutations or deletions in the p53 gene may contribute to cancer initiation and progression. Notably, the p53 gene is widely expressed in the brain, where it participates in various cellular processes, including dendritogenesis, oxidative stress response, cell death, autophagy, DNA repair, and cell cycle arrest (Lei et al., 2022). Therefore, it has been termed the guardian of the genome. Notably, accumulating evidence reveals a close relationship between p53 and ferroptosis. As a key regulator, p53 can modulate intracellular iron metabolism by regulating related genes. In lipid metabolism, p53 can affect ferroptosis susceptibility through influencing lipid synthesis, degradation, and transport, thereby altering the composition and fluidity of the cell membrane. Furthermore, p53 can regulate both ROS production and clearance, as well as amino acids metabolism through multiple mechanisms, highlighting its important role in modulating ferroptosis (Ji et al., 2022; Liu and Gu, 2022). Given the broad neurobiological functions of p53 described above, it is plausible that p53-mediated pathways may also play a significant role in the pathophysiology of PTSD. For example, the p53-mediated regulation of iron metabolism and ROS production could potentially influence the oxidative stress and neuronal damage observed in PTSD. Further studies have indicated that the ROS-JNK-p53 pathway is involved in neuronal apoptosis in PTSD models. *In vitro* experiments have demonstrated that electrical stimulation triggers neuronal apoptosis through activating the ROS/JNK/p53 signaling pathway. However, the antioxidant N-acetylcysteine (NAC) can attenuate ROS generation, suppresses JNK/p53 activation, and decrease the apoptotic rate in HT-22 cells. These findings imply that the ROS-JNK-p53 pathway may serve as a critical mediator of neuronal apoptosis in PTSD (Lv et al., 2024), highlighting the exploration of p53-mediated ferroptosis as a promising avenue for novel therapeutic strategies.

## 6 Therapeutic strategies targeting ferroptosis in PTSD

Several ferroptosis modulators have been explored for their possible therapeutic benefits in PTSD treatment (Table 2).

**TABLE 2:** Therapeutic interventions targeting ferroptosis in PTSD.

Intervention	Mechanism	Potential effect	References
NAC	Antioxidant, scavenges ROS, inhabits ferroptosis	Reduces oxidative stress, alleviates PTSD symptoms	Maier et al., 2020
Tempol	Antioxidant, modulates catalase and SOD activity, maintains GSH/GSSG ratio	Prevents lipid peroxidation, alleviates PTSD symptoms	Alzoubi et al., 2018b
EDA	Suppresses oxidative stress and neuroinflammation	Alleviates depression	Dang et al., 2022
NaHS	Antioxidant	Alleviates anxiety and depression symptoms, reduces iron accumulation	Wang et al., 2021
Cerebrolysin	Maintains GSH, GSSG and GSH/GSSG ratio, prevents oxidative damage	Reduces oxidative stress, lowers ferroptosis risk	Alzoubi et al., 2018b
Taurine	Antioxidant, inhibits ferroptosis	Mitigates oxidative stress, improves mitochondrial function	Bhattacharjee et al., 2021
Vitamin E	Reduces oxidative stress biomarkers, protects neurons	Reduces oxidative stress, prevents ferroptosis, supports neuronal survival and function	Ahmed et al., 2020
Etazolate	Regulates GSH, GSSG, GPX4 and TBARs levels	Mitigates oxidative stress, antidepressant effects	Alzoubi et al., 2019
Acupuncture	Increases Nrf2, HO-1 and BDNF levels, inhibits ferroptosis	Reduces oxidative stress and improves PTSD symptoms	Zhou et al., 2019; Chen et al., 2024

NAC, N-acetylcysteine; EDA, edaravone; NaHS, sodium hydrosulfide; BDNF, brain derived neurotrophic factor; GSH, glutathione; GSSH, glutathione disulfide; SOD, superoxide dismutase; GPX, glutathione peroxidase; ROS, reactive oxygen species; GPX4, glutathione peroxidase 4.

### 6.1 free radical scavenger

As a thiol compound and a precursor to L-cysteine and reduced GSH, NAC functions as a free radical scavenger. Specifically, it reduces oxidative stress by neutralizing ROS and protecting cells from oxidative damage (Li J. et al., 2022). This is particularly pertinent in PTSD, where oxidative stress is frequently implicated in the pathophysiology of the disorder. Beyond its antioxidant role, NAC may also inhibit ferroptosis by activating Nrf2, which regulates the expression of metallothioneins, ferritins, and ferroportin to prevent iron accumulation (Kerins and Ooi, 2018). Notably, treatment with NAC has been shown to alleviate symptoms in veterans diagnosed with PTSD (Maier et al., 2020). Further research has confirmed that NAC treatment significantly improves cognitive function and reduces apoptosis of hippocampal neurons in PTSD model mice (Zhou et al., 2025). Collectively, these findings indicate that NAC may effectively counteract the oxidative pathologies associated with PTSD, thereby offering a promising avenue for this debilitating condition. Similarly, Tempol (4-hydroxy-2,2,6,6-tetramethylpiperidine-N-oxyl), a nitroxide radical compound that has demonstrated potent antioxidant properties in PTSD models. Specifically, it modulates catalase and SOD activity while maintaining reduced GSH-to-glutathione disulfide (GSH/GSSG) ratio (Alzoubi et al., 2018b). This holds significant importance in the context of ferroptosis, as maintaining the GSH/GSSG ratio is crucial for preventing lipid peroxidation and the cell death associated with ferroptosis. Furthermore, edaravone (3-methyl-1-phenyl-2-pyrazolin-5-one, EDA), another free radical scavenging, has been shown to alleviate anxiety and depressive-like behaviors in mouse models by regulating oxidative stress and neuroinflammation (Dang et al., 2022). Given that PTSD is a psychiatric disorder characterized by anxiety and depression, edaravone may provide therapeutic benefits for PTSD patients.

Evidence indicates that GPX4 is crucial for protecting cells against ferroptosis by reducing lipid peroxides (Zhang et al., 2024). Additionally, the solute carrier family 7 member 11 (SLC7A11) also plays a key role in regulating the cystine-glutamate antiporter system, which maintains intracellular GSH levels and effectively prevents ferroptosis (Wang et al., 2023). Notably, sodium hydrosulfide (NaHS), a prominent scavenger of free radicals, has been shown to alleviate anxiety and behaviors symptoms. This effect is probably linked to its capacity to reduce iron accumulation and oxidative stress while enhancing the expression of GPX4 and SLC7A11 (Wang et al., 2021). Given the persistent oxidative stress, iron metabolism abnormalities, and anxiety or depression symptoms in PTSD patients. NaHS may represent a novel and effective strategy for the treatment of PTSD, warranting further investigation in clinical settings. Furthermore, strategies involving iron chelators and free radical scavengers have emerged as promising approaches to inhibit ferroptosis. Therefore, these strategies may potentially improve therapeutic efficacy, thereby providing a more effect strategies for PTSD treatment.

### 6.2 Antioxidants

Therapies targeting oxidative stress have shown significant potential in alleviating PTSD symptoms. For example, cerebrolysin is a mixture of low molecular weight neuropeptides sourced from porcine brain, and it has been demonstrated to effectively prevent abnormal alterations in GSH, GSSG, and the GSH/GSSG ratio in PTSD models induced by SPS (Alzoubi et al., 2018b). Given that ferroptosis is driven by oxidative stress-mediated lipid peroxidation, cerebrolysin’s ability to sustain antioxidant homeostasis could potentially reduce the risk of ferroptosis in PTSD. Similarly, taurine exerts therapeutic effects through its antioxidant properties, which help stabilize the mitochondrial membrane potential and influence the mitochondrial permeability transition pore (mPTP) (Palmi et al., 2000). Additionally, research in PTSD models has shown that treatment with taurine can improve mitochondrial function (Bhattacharjee et al., 2021). Given that mitochondrial dysfunction plays a significant role in both inducting oxidative stress and progressing ferroptosis, taurine’s role in maintaining mitochondrial health may help mitigate oxidative stress and thereby inhibit ferroptosis.

Moreover, vitamin E, a fat-soluble vitamin that readily crosses the blood-brain barrier (BBB), has shown potential in reducing oxidative stress (Miyazawa et al., 2019). Studies have indicated that treatment with vitamin E can prevent cognitive decline in individuals affected by various neurodegenerative disorders associated with ferroptosis, including PD and AD (La Fata et al., 2014; Yang et al., 2017). In individuals with PTSD, vitamin E has also been found to effectively lower oxidative stress biomarkers, including the decreased GSH/GSSG ratio and diminished activity of GPX and catalase. Additionally, it protects against the decline in global histone 3 acetylation and antioxidant defense gene named brain derived neurotrophic factor (BDNF) levels (Ahmed et al., 2020). Notably, histone acetylation is an important epigenetic modification that has been shown to regulate the expression of ferroptosis-related genes, such as GPX4 and SLC7A11, thereby reducing the sensitivity of cells to ferroptosis and playing a crucial role in regulating this cell death process (Yang et al., 2023). These findings suggest that vitamin E may exert its therapeutic effects in PTSD by preventing ferroptosis and preserving neuronal survival and function. Additionally, phosphodiesterase-4 inhibitors have garnered attention for their ability to reduce oxidative stress and the risk of ferroptosis by increasing intracellular cAMP levels. Etazolate, a selective phosphodiesterase-4 inhibitor specific for cAMP, has garnered attention for its anxiolytic and antidepressant effects. Research has confirmed that treatment with etazolate can effectively restore the levels and activities of GSH, GSSG, GPX, and TBARs in PTSD models (Alzoubi et al., 2019). This restoration suggests that etazolate may mitigate oxidative stress and thereby reduce the risk of ferroptosis, contributing to its therapeutic potential in PTSD. In summary, antioxidants such as Cerebrolysin, taurine, vitamin E, and etazolate have shown promise in reducing the risk of ferroptosis in PTSD by mitigating oxidative stress, maintaining antioxidant balance, and preserving mitochondrial health. These findings offer new insights into the treatment of PTSD and provide valuable references for future research and clinical applications.

### 6.3 Acupuncture

Traditional Chinese Medicine (TCM) encompasses a range of therapeutic approaches, including herbal medicines, acupuncture, and moxibustion, and is known for its multi-pathway, multi-target, and high safety profile (Zhou et al., 2024). Among these, acupuncture, an external therapeutic approach, has been proven effective for various conditions. One important modality of acupuncture therapy is electroacupuncture, which combines traditional acupuncture with modern electrical stimulation to enhance therapeutic effects. Specifically, electroacupuncture has been shown to promote the nuclear translocation of Nrf2 within neurons (Chen et al., 2024). Notably, Nrf2, a key transcription factor, is crucial for coordinating antioxidant responses and significantly contributes to the reduction of ferroptosis (Ma, 2013). Specifically, Nrf2 not only modulates the expression and functionality of GPX4 but also regulates the expression of genes encoding proteins essential for GSH synthesis, including System Xc^–^ and the catalytic/modulatory subunits of glutamate-cysteine ligase (GCLC/GCLM), and glutathione synthetase (GSS). Importantly, elevated Nrf2 levels enhance xCT expression and modify GPX4 synthesis and function, ultimately inhibiting ferroptosis (Anandhan et al., 2020). Research has indicated that electroacupuncture treatment can effectively inhibit ferroptosis by activating Nrf2, increasing protein expression of solute carrier family 7 member 11 (xCT) and GPX4, thereby reducing cerebral ischemia/reperfusion injury (Zhu et al., 2024). Similar in PTSD, pretreatment with electroacupuncture provides therapeutic benefits for PTSD by increasing Nrf2, HO-1, and BDNF levels, thereby activating antioxidant pathways (Zhou et al., 2019). However, these therapeutic benefits of electroacupuncture pretreatment are negated when Nrf2 is knocked down in the hippocampus. These findings suggest that electroacupuncture’s therapeutic benefits may be mediated through its protective effects against ferroptosis. Further research is needed to fully elucidate the underlying mechanisms and explore the potential clinical applications of electroacupuncture in treating PTSD and other neurodegenerative disorders.

## 7 Conclusion and future perspective

In this review, we analyzed numerous studies focusing on the role of ferroptosis in PTSD. This hypothesis that ferroptosis might contribute to the intricate pathogenic processes of PTSD is supported by some critical findings. Firstly, the ACSL4, ACO1, and GSS genes have been verified to serve as two key functions: they are not only key predictors of PTSD risk but also play essential roles in the development of the disorder. Secondly, the hallmark features of ferroptosis, including iron dysregulation, lipid peroxidation, neuroinflammation as well as mitochondrial dysfunction, are clearly observed in PTSD patients. Additionally, many therapeutic approaches for PTSD have been found to exhibit anti-ferroptotic effects. Based on these established associations, we encourage more in-depth research to further explore the interplay between ferroptosis and PTSD.

Although there is increasing attention on ferroptosis and its possible involvement in PTSD, the existing literature has certain methodological limitations that require to be addressed. Firstly, most of the existing studies rely on animal models to explore the mechanisms underlying ferroptosis in PTSD. While these models offer valuable insights, they may not fully capture the complexity of PTSD in humans. Therefore, these findings from animal models should be interpreted with caution and validated in human studies. Moreover, the use of different animal species and genetic backgrounds can introduce variability in the results. This variability can complicate the interpretation of results and the generalizability of findings across different studies. In conclusion, while the current literature provides a foundation for understanding the potential role of ferroptosis in PTSD, significant gaps remain. Addressing the methodological limitations of existing studies will be crucial for advancing our knowledge and developing effective therapeutic strategies for PTSD.
